# Supplementary material for: Single-Cell Transcriptomics Reveals the Complexity of the Tumor Microenvironment of Treatment-Naive Osteosarcoma
Source: Front Oncol. 2021 Jul 21;11:709210. doi: 10.3389/fonc.2021.709210 (PMC8335545; doi:10.3389/fonc.2021.709210)
Supplement: Supplementary Document 1 — Kaplan-Meier curve of the 518 genes associated with survival. [file DataSheet_1.zip › Supplementary Document 1/sur.MGST2.pdf]

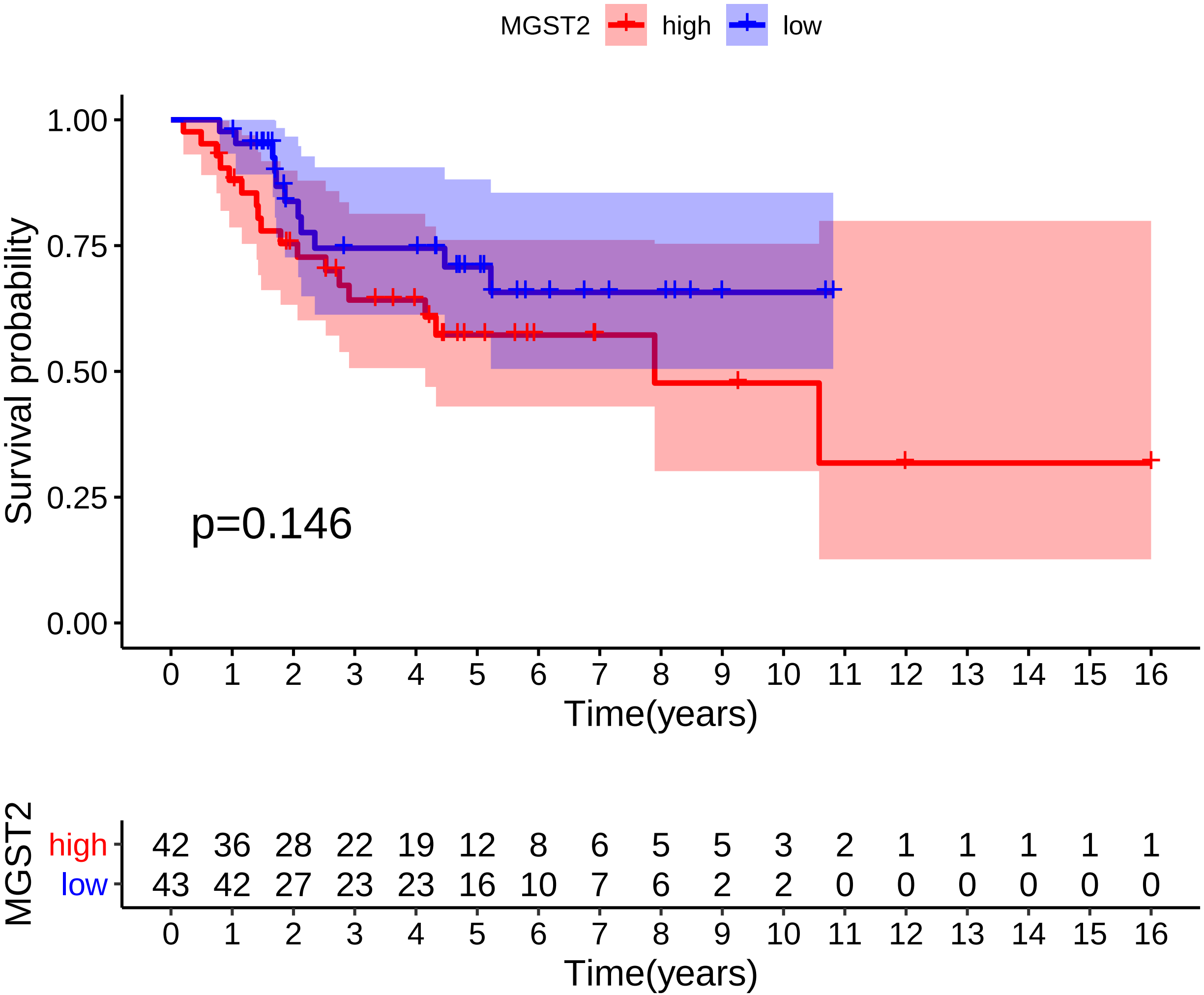

MGST2
high
low
Survival probability
1.00
0.75
0.50
0.25
0.00
p=0.146
0
1
2
3
4
5
6
7
8
9
10
11
12
13
14
15
16
Time(years)
MGST2
high
low
42 36 28 22 19 12 8 6 5 5 3 2 1 1 1 1 1
43 42 27 23 23 16 10 7 6 2 2 0 0 0 0 0 0
Time(years)
